# The Chimeric Myo-Osseous Medial Femoral Condyle Flap for Tibial Nonunion: A Case Report

**Published:** 2018-07-10

**Authors:** Erwin A. Kruger, Oded Ben-Amotz, Shaun D. Mendenhall, L. Scott Levin

**Affiliations:** ^a^Department of Orthopaedic Surgery; ^b^Division of Plastic Surgery, Department of Surgery, University of Pennsylvania, Philadelphia

**Keywords:** medial genicular artery flap, medial femoral condyle flap, vascularized bone flap, chimeric free flap, myo-osseous flap

## Abstract

**Objective:** The descending genicular artery provides the dominant pedicle for the medial femoral condyle and medial femoral trochlear free flaps. Variations of the flap include using a skin paddle for monitoring, a vastus medialis muscle component, and the medial superior geniculate artery for the pedicle. We present a case of a 49-year-old man with a distal tibial fracture from a motor vehicle accident complicated by chronic osteomyelitis, poor soft tissue envelope, and tibial nonunion. **Methods:** The composite lower extremity defect was reconstructed with a chimeric myo-osseous variant of the medial femoral condyle free flap since no perforator was available to the skin and there was a large amount of dead space. **Results:** At 9 months postoperatively, there was successful tibial union, adequate coverage of the defect, and a return to unassisted ambulation. **Conclusions:** The chimeric myo-osseous variant of the medial femoral condyle free flap adds to the versatility of this flap and has application in reconstructing defects of bone that also require soft tissue coverage.

## OBJECTIVE

The medial genicular artery system offers a robust blood supply for corticoperiosteal free flaps harvested from the medial femoral condyle (MFC).^1^^-^3 Applications of this flap include treatment of upper and lower extremity bony nonunions and segmental mandibular reconstruction.^1^^-^3 Two variations are the MFC flap and the medial femoral trochlear flap, which have gained popularity in a variety of bone nonunions.^4^^-^10 The dominant pedicle is the descending geniculate artery (DGA) that comes off the superficial femoral artery, although the medial superior geniculate artery that comes off the popliteal artery can serve as an alternative source vessel to the flap. The MFC osteocutaneous flap has also been well described that adds a small skin paddle for monitoring.^11^

A recent systematic review of the literature of these flaps identified 16 recipient sites in the upper extremity and the lower extremity.^12^ The data from the upper and lower extremity experience resulted in a 97.8% union rate with minimal complications.^12^ This experience suggests these flaps to be versatile and suitable for an array of small osseous defects, malunions, and nonunions.

In this case report, we describe a chimeric myo-osseous MFC flap incorporating the osseous component of the MFC flap with a segment of the vastus medialis nourished by a perforator from the descending genicular arterial system for a combined tibial nonunion with a poor soft tissue envelope.

## METHODS

**Figure 1:**
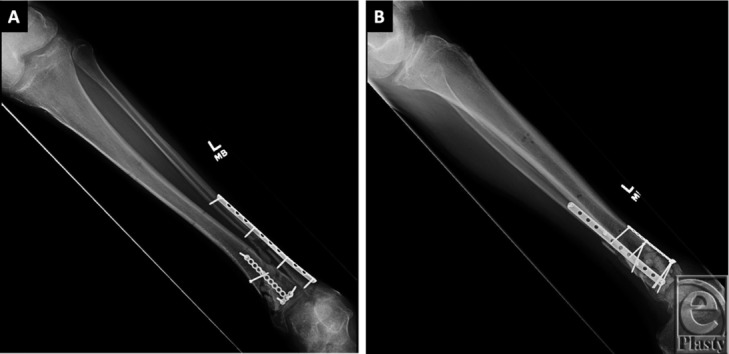
(a, b) Anteroposterior and lateral radiographs of left tibia and fibula showing bridging plates with screws transfixing visible fractures of the distal tibia and fibula. There are mildly displaced butterfly fracture fragments of both bones. There is no radiographic evidence of bony bridging of the fracture fragments.

**Figure 2:**
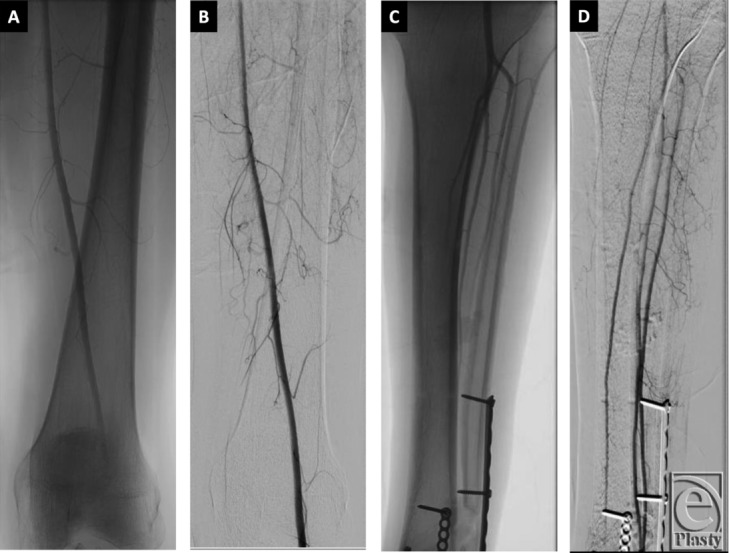
Arteriogram views of the supracondylar region (a, b) and the distal lower extremity (c, d) showing the widely patent common femoral, superficial femoral, and popliteal arteries. There is 3-vessel runoff to the foot via the widely patent anterior tibial, posterior tibial, and peroneal arteries.

A 49-year-old police officer sustained a comminuted fracture of the distal tibia and fibula following a motor vehicle accident. This was managed by open reduction and internal fixation, and complicated by fibular nonunion, hardware failure, and chronic osteomyelitis of the tibia. He underwent multiple debridements and revision procedures and presented with suspected chronic hardware colonization despite placement of antibiotic beads. The preoperative radiographs are shown in Figure 1. Angiogram of his involved extremity demonstrated 3-vessel runoff (Fig 2). The surgical plan included removal of tibial hardware and antibiotic beads (previously placed), debridement of the sinus tract, bone debridement, and new antibiotic bead placement, interval intravenous antibiotics, followed by a free vascularized MFC bone flap to the distal tibial defect.

The patient underwent 2 operations with the orthoplastic reconstructive team. In the first stage, the tibial spanning plate was removed without difficulty. The distal cortex adjacent to the antibiotic bead cavity was white and chalky compared with the viable proximal diaphyseal bone. A sequestrectomy was performed after the antibiotic beads were removed. The lateral wall of the tibia appeared healed and consolidated with paprika sign observed. The medullary canal was expanded. The resulting bone defect measured 3 × 3 × 4 cm, which was approximately 320° of the distal tibial shaft circumference.

**Figure 3:**
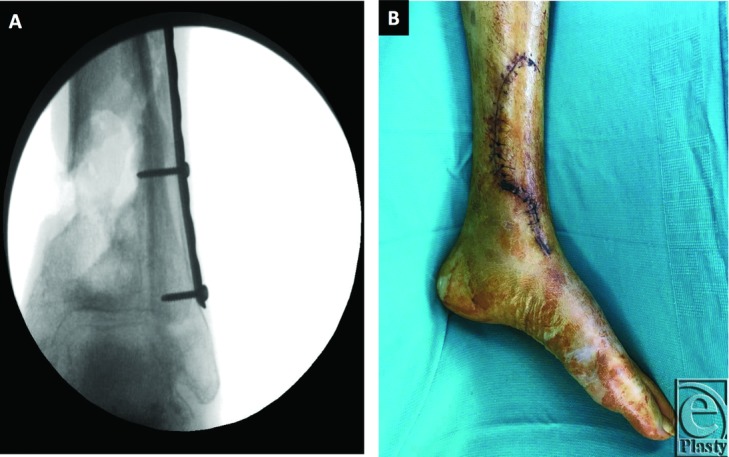
(a, b) Fluoroscopic image and wound following first-stage debridement and antibiotic bead placement.

All granulation and nonviable tissues surrounding the tibia were removed. Adjacent tissue rearrangement was performed using a back cut on the medial face of the tibia with the skin flap that was raised on the basis of the original incision. The flap was then advanced, and the wound was closed over fresh antibiotic beads. Intraoperative cultures grew rare methicillin-resistant *Staphylococcus aureus*. Infectious Disease was consulted, and the patient was given long-term intravenous vancomycin for the treatment of osteomyelitis. Postoperative radiographs and photographs after the first stage are shown in Figure 3.

**Figure 4:**
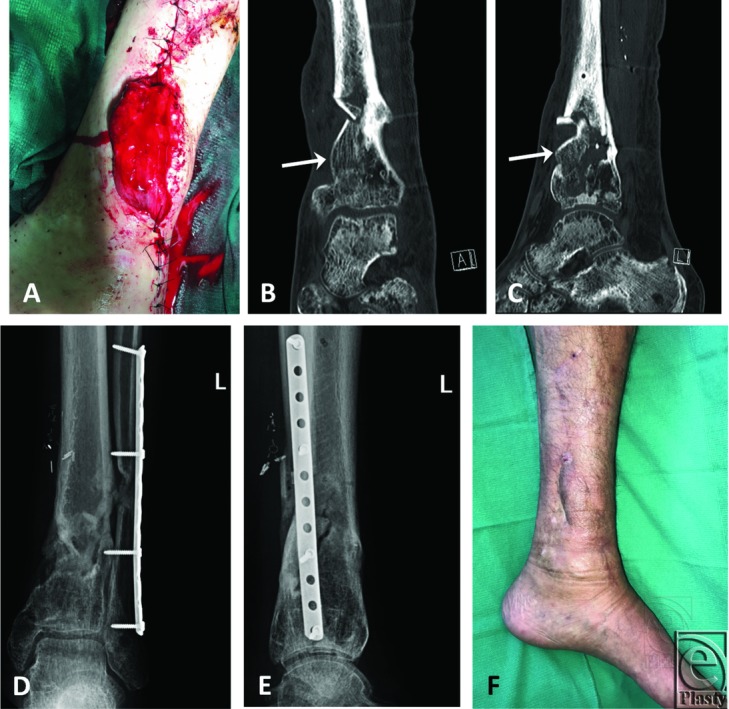
Immediate postoperative view of the chimeric myo-osseous MFC flap (a), AP (b), and lateral (c) computed tomographic scans of the MFC flap (arrow) at 3 months postoperatively, AP (d) and lateral (e) plain radiographs of the flap at 9 months postoperatively showing good bone healing, and a final photograph of the healed flap at 9 months postoperatively (f). MFC indicates medial femoral condyle; AP, anteroposterior.

The second operation took place 4 weeks later. The original incisions were opened, and the antibiotic beads were removed. New bone and soft tissue cultures were obtained and sent for Gram stain, acid-fast bacilli, fungal, aerobic, and anaerobic cultures. The posterior tibial neurovascular bundle was identified. Attention was then turned to raising the MFC flap. A longitudinal incision was made over the left knee and the fascia of the vastus medialis was incised. The vastus muscle was retracted anteriorly. The DGA was identified distally as it approached the medial face of the diaphysis of the MFC. Retrograde dissection was carried out, and muscle perforators to the vastus medialis were identified and preserved. There were no direct perforators coming off the descending geniculate that could be used for a skin paddle, so the muscle was chosen for the composite flap to help fill the dead space and to reconstruct the soft tissue envelope. We used an oscillating saw to harvest a 1 × 3 × 3.5-cm bone block from MFC cortex that included cortical-cancellous bone and periosteum. The bone flap was trimmed and impacted into the medial tibial defect, osteotomizing the undersurface of the flap and bending it so that a gentle curve could exist from the medial native tibia to the cortical-cancellous graft distally. No fixation of the flap was needed, as it was stable within the defect. Microvascular anastomosis was performed end-to-end with 9-0 nylon between the posterior tibial artery and the DGA, resulting in excellent flow. Three-millimeter microvascular couplers were then used to couple 2 veins, resulting in excellent outflow and Doppler signals. The muscle was advanced and placed over the chronic wound area above the periosteum of the bone component of the composite flap (Fig 4). A 12/1000 of an inch skin graft was applied over the muscle. There were no postoperative complications and routine healing took place. The intraoperative Gram stain was negative, as were all the final cultures.

## RESULTS

At 9 months, the intercalary defect of his tibia was bridged with the MFC flap without any evidence of lucency by plain radiographs. He was able to plantarflex his ankle to 60°. Mild lower extremity edema was managed by compression stocking, and the patient was able to ambulate full weight-bearing without the use of a cane or walker. The healed flap, radiographs, and computed tomographic scans of his bony bridging are shown in Figure 4.

In this particular case, other options including nonvascularized cancellous autografts, demineralized bone matrix, and bone morphogenic protein would have been insufficient because of defect size, its chronically infected state, and the need of a well-vascularized soft tissue envelope. The free vascularized bone and the muscle flap proved successful for reconstruction of this complex composite defect.

## CONCLUSIONS

The MFC region as a donor site for free flaps has had an increasing body of literature for use in reconstructive microsurgery. Historically, Acland et al^13^ introduced a free neurovascular skin flap based on the saphenous branch of the descending genicular artery. Hertel and Masquelet^14^ described a regional reverse flow osteoperiosteal flap pedicled on the saphenous branch. Martin et al^3^ described an osteocutaneous variant of this flap using the DGA. Doi and colleagues^1^^,^^2^ published on the vascularized periosteal bone flap variant using the DGA as a free flap as well. Since these landmark descriptions and clinical case reports, many studies of the MFC flap have been reported.^11^^,^^15^^,^^16^

We report use of a combined myo-osseous flap for the treatment of a complex soft tissue defect and underlying chronic tibial osteomyelitis. Our case report is preceded by an excellent cadaveric study and case report by Rahmanian-Schwarz et al.^17^ These investigators used 11 fresh adult cadavers to analyze the characteristics and consistency of the DGA along with its branching pattern to the vastus medialis and reported 2 clinical cases of a combined osteomyocutaneous flaps for calcaneal reconstruction.^17^ This study confirmed the reliability of a muscular branch of the DGA that can be used for combined flaps of this region.^17^ Our report builds on this experience, confirms that a large piece of vastus medialis can be utilized with the flap without a skin paddle, and expands the use of medial genicular artery chimeric flaps for complex reconstruction of the extremities.

This study reaffirms that anatomic variations of the DGA exist, as others have described and presented as a potential disadvantage in previous cadaveric studies.^15^ In addition, others have commented on the relatively short pedicle length and relatively small diameter of the vessels as a consideration in presurgical planning.^15^^,^^16^ However, this has not been a problem in our experience when the entire length of the pedicle is taken back to the superficial femoral artery. The advantages of the flap are many: relatively easy dissection and harvest; a blood supply sparing other structures of the lower limb; and now a growing experience with combined tissue flaps. The MFC flap has become a versatile flap in the armamentarium of reconstructive microsurgery.

The MFC flap can be used as myo-osseous flap for complex bony reconstruction when soft tissue coverage or augmentation is required. Anatomic variations exist, and individual cases should be assessed intraoperatively for reliable perforators to consider myo-osseous or osteomycocutaneous chimeric flaps. This flap will continue to be an asset to reconstructive microsurgeons, especially those involved in orthoplastic reconstruction.
